# Fenretinide stimulates redox-sensitive ceramide production in breast cancer cells: potential role in drug-induced cytotoxicity

**DOI:** 10.1038/sj.bjc.6602212

**Published:** 2004-10-26

**Authors:** F Rehman, P Shanmugasundaram, M P Schrey

**Affiliations:** 1 Section of Endocrinology & Metabolic Medicine, Imperial College London, St Mary's Hospital, Praed Street, London W2 1NY, UK

**Keywords:** 4HPR, ceramide, MCF-7

## Abstract

The synthetic retinoid *N*-(4-hydroxphenyl) retinamide (4HPR) has manifold actions, which may contribute to its chemopreventive effects on breast cancer cell growth and progression. A role for ceramide as a stress-response signal is investigated here during the cytotoxic action of 4HPR in MCF-7 cells. *N*-(4-hydroxphenyl) retinamide induced a dose-dependent decline in cell growth and survival associated with a maximal 10-fold increase in ceramide production at 10 *μ*M. *N*-(4-hydroxphenyl) retinamide exhibited a greater potency than all-*trans* retinoic acid (ATRA) on growth inhibition and ceramide production. The synthetic peroxisome proliferator-activated receptors agonist troglitazone (TGZ), but not the native ligand 15-deoxy-delta 12,14-prostaglandin J_2_, abrogated both these actions of 4HPR but not that of ATRA. The antioxidant *N*-acetylcysteine mimicked the abrogative effect of TGZ on 4HPR action, while the exogenous oxidant H_2_O_2_ also stimulated ceramide production. The inhibitors of *de novo* ceramide synthesis, fumonisin B_1_ and myriocin, blocked the ceramide response to 4HPR and partially reversed the apoptotic response, but did not prevent the overall decline in cell survival. The pancaspase inhibitor Z-VAD fmk reduced the decrease in cell survival caused by 4HPR, but did not affect the ceramide response. These findings describe a novel redox-sensitive elevation of ceramide levels associated with the cytotoxic response of breast cancer cells to 4HPR. However, a major mediatory role for this sphingolipid in this context remains equivocal.

The potential application of retinoids for breast cancer prevention and treatment reflects their important role as regulators of cell growth and differentiation. Retinoids initiate many of their actions by ligand-induced dimerisation of retinoic acid receptors (RARs) and retinoid X receptors, followed by receptor binding to retinoid response elements on DNA and transactivation of retinoid-response target genes. All-*trans* retinoic acid (ATRA), the most potent naturally occurring metabolite of vitamin A, reversibly inhibits the growth of hormone-dependent human breast cancer cells, requiring the activation of RAR*α*-mediated gene transcription for this effect (Dawson *et al*, 1995). Synthetic modification of the carboxyl end of retinoic acid with an *N*-4 hydroxyphenyl group results in the formation of *N*-4-(hydroxyphenyl) retinamide (4HPR) or fenretinide. *N*-4-(hydroxyphenyl) retinamide is more potent than ATRA, both as an antiproliferative agent and inducer of apoptosis in the majority of cancer cell lines tested (Oridate *et al*, 1996; Zou *et al*, 1998). Compared with ATRA, 4HPR exhibits reduced hepatotoxicity and increased efficacy in inhibiting mammary carcinogenesis in animal models (Ulukaya and Wood, 1999). Recent clinical studies also highlight a potential chemopreventative action of 4HPR on breast cancer recurrence in high-risk premenopausal women (Veronesi *et al*, 1999; Decensi *et al*, 2000).

Although 4HPR can transactivate certain retinoid receptors and RAR antagonists can partially block 4HPR-induced apoptosis (Sun *et al*, 1999) compared with ATRA, 4HPR binds with low affinity to RAR and demonstrates poor transactivation of RAR/RXR response elements in human breast cancer cells (Sheikh *et al*, 1995). Retinoid receptors can also interact with peroxisome proliferator-activated receptors (PPARs) either directly through the formation of heterodimers (Schulman *et al*, 1998) or indirectly by regulating their mutual expression (Stoll, 2002; James *et al*, 2003). In this regard, when combined with PPAR*γ* agonists such as troglitazone (TGZ), ATRA synergistically and irreversibly inhibits growth and induces apoptosis in human breast cancer cells (Elstner *et al*, 1998). Whether PPAR*γ* agonists manifest similar interactions with 4HPR in breast cancer cells is unknown.

As well as activating receptor-mediated mechanisms, retinoids such as 4HPR may modulate cell function via alternative receptor-independent pathways involving cellular signals such as reactive oxygen species (ROS) (Sun *et al*, 1999) and the sphingolipid ceramide (DiPietrantonio *et al*, 1998).

Evidence supporting a role for ceramide during 4HPR action on HL-60 leukaemia cells and neuroblastoma cells is based on studies demonstrating an elevation of cellular ceramide levels in response to 4HPR and the ability of the ceramide synthase inhibitor fumonisin B_1_ (FB_1_) to block this ceramide response and prevent 4HPR-induced apoptosis (DiPietrantonio *et al*, 1998; Maurer *et al*, 1999). However, in other instances where induction of apoptosis is accompanied by an elevation of ceramide, FB_1_ failed to prevent the apoptotic response while blocking ceramide production (Nagy *et al*, 2000; Miguet *et al*, 2001).

Several studies have also demonstrated a role for ROS during the induction of apoptosis by 4HPR in various cell types (Sun *et al*, 1999; Suzuki *et al*, 1999). Furthermore, cellular redox status and ROS are also known to regulate critical steps in a wide variety of cellular functions including sphingolipid pathways involved in stress-response signalling (Andrieu-Abadie *et al*, 2001). The nature and relevance of ceramide production and signalling, and its relationship to redox status during 4HPR action in human breast cancer cells, are unknown.

A better understanding of the molecular mechanisms involved in the cytotoxic actions of anticancer agents such as 4HPR is crucial for the further development and chemotherapeutic use of such drugs. In the present study with the human breast cancer cell line MCF-7, we have compared the effects and interactions of both native and synthetic retinoids (ATRA and 4HPR) and PPAR*γ* agonists (15-deoxy-delta 12,14-prostaglandin J_2_, 15d-PGJ_2_ and TGZ) on cell growth, survival and ceramide production. Under a variety of conditions, retinoid action on cell growth, survival and cytotoxicity was associated with corresponding changes in ceramide production. *N*-(4-hydroxphenyl) retinamide exhibited much greater potency and efficacy than ATRA on inhibiting cell growth and inducing ceramide production and cytotoxicity. Troglitazone, but not 15d-PGJ_2_, blocked the antiproliferative and cytotoxic actions of 4HPR and reduced the ceramide response, possibly as a consequence of its antioxidant properties. Although FB_1_ prevented the ceramide response to 4HPR, the decline in cell survival was unaffected.

## MATERIALS AND METHODS

### Materials

Unless otherwise stated, all sphingolipid standard chemicals, reagents and materials for cell culture were purchased from Sigma (Poole, Dorset, UK). Culture media and supplements were obtained from ICN Biochemicals (High Wycombe, UK). 15-deoxy-delta 12,14-prostaglandin J_2_ was obtained from Affiniti Research Products (Exeter, UK), TGZ was a gift from Glaxo Wellcome (Uxbridge, UK) and SDZ PSC 833 was a gift from Novartis Pharma AG (Basel, Switzerland).

### Cell culture

MCF-7 cells were obtained from the European Collection of Animal Cell Cultures (Salisbury, UK) and maintained at 37°C in Eagle's minimal essential medium (EMEM) supplemented with glutamine (2 *μ*M), nonessential amino acids and 10% foetal calf serum. For all experiments monitoring cell growth, apoptosis and ceramide production cell stocks grown in 25 cm^2^ flasks were trypsinised and seeded into six-well plates in supplemented EMEM containing penicillin (100 U ml^−1^), streptomycin (100 *μ*g ml^−1^) and amphotericin-*β* (250 ng ml^−1^).

### Cell growth

The effects of various treatments (see legends for details) on MCF-7 cell growth were monitored over a 72 h period. Trypsinised cells were seeded into six-well plates and allowed to plate down for 18 h prior to addition of treatments. Cell numbers were determined at the end of the growth period by counting isolated cell nuclei by the Coulter principle following treatment of intact cells with Zaponin (Coulter, Luton, UK).

### Cell survival

The effects of various treatments on MCF-7 cell survival was monitored after a 72 h incubation period using the Promega MTS assay (Madison, WI, USA) as an index of cell viability. The tetrazolium compound MTS is bioreduced by cells into a coloured formazan product that absorbs light at 490 nm. Absorbance is proportional to the number of viable cells present. Trypsinised cells were diluted to a concentration of 10^5^ cells ml^−1^ and 50 *μ*l aliquots were seeded into 96-well plates (5000 cells well^−1^). After a 6 h plating period, various treatments were added in 50 *μ*l and the cells incubated for a further 72 h in EMEM containing supplements. After this incubation, 20 *μ*l of MTS assay reagent was added and absorbance measured at 490 nm after 90 min of colour development. After subtracting the background absorbance of cell-free incubations, cell survival was determined by expressing all values as a percentage of control absorbance measured in the absence of treatment.

### DNA fragmentation ELISA

This photometric enzyme-immunoassay measures cytoplasmic histone-associated DNA fragments (mono- and oligonucleosomes), which are generated during apoptosis (Roche Applied Science). The enrichment of nucleosomes in the cytoplasm of treated cells is expressed as a fold induction of apoptosis compared to untreated controls. Assay samples were prepared according to the manufacturer's instructions (Roche Applied Science, Mannheim, Germany). MCF-7 cells were seeded in supplemented EMEM into 96-well plates at 10^4^ cells well^−1^. After 18 h, treatments were added (see legend Figure 6 for details) and the cells incubated for a further 24 h. The medium was then removed and cell lysates were prepared and centrifuged for 10 min at 200 **g**. A measure of 20 *μ*l aliquots of the supernatant (cytoplasmic fraction) was then assayed for nucleosome content by ELISA in accordance with the manufacturer's protocol.

### Sphingolipid metabolism

Ceramide and glucosylceramide were measured by radiolabelled incorporation of [^3^H] palmitic acid into the appropriate sphingolipids, as described previously (Cabot *et al*, 1998). MCF-7 cells were grown to approximately 70% confluence in six-well plates in 2 ml of supplemented EMEM. At this point, the fresh medium was added containing the various treatments (see legends for details) and [^3^H]palmitic acid (1 *μ*Ci ml^−1^) (Amersham, UK). The cells were then incubated for a further 24 h. Experiments were terminated by the removal of the medium and addition of 2 ml acidified methanol (2% acetic acid). Any non-adherent cells were retrieved by centrifugation. Cellular lipids were allowed to be extracted into the methanol over a 30 min period at room temperature. This methanol extract was removed to glass tubes and the lipids extracted into a chloroform phase following addition and mixing with 2 ml chloroform and 2 ml of KCl/EDTA (2 M/5 mM). Total cellular radiolabelled lipid was measured by counting a 100 *μ*l aliquot of this chloroform extract following solvent evaporation. The ceramide standard containing primarily stearic and nervonic species (C_18_ and C_24_) was added to a 1 ml aliquot of each lipid extract sample to act both a carrier and marker. These ceramide species were separated from the remaining lipids by TLC in the solvent system chloroform/acetic acid (90 : 10,v v^−1^). C_18_ and C_24_ ceramide were also resolved from one another in this system with Rf values of 0.29 and 0.38, respectively. However, this system does not resolve the biologically inactive dihydroceramides. Glucosylceramide was resolved from other lipids by TLC in the solvent system chloroform/methanol/ammonia (70 : 20 : 4, v v^−1^) also using appropriate commercial standards, as described above. Resolved lipid bands were visualised by staining with iodine vapour, scraped into scintillation vials containing 0.5 ml water and 10 ml ECOSCINT, and radioactivity measured by liquid scintillation counting. The majority of [^3^H]palmitic acid incorporated into cellular lipids was present as phospholipid esters. These polar lipids remained at the origin in the solvent systems used and in no instances where changes in ceramide production were observed in response to 4HPR, were there significant accompanying changes in total phospholipid. Similarly, no significant changes in total cellular lipids were observed in response to any experimental treatments. Values for radiolabelled incorporation into ceramide and glucosylceramide are expressed as a percentage of total cellular lipid. Such ‘normalisation’ of the data will compensate for any skewness in the data, which would occur if any treatments also affected general permissive lipid pathways such as fatty acid uptake or CoA ester formation. Similarly, elongation, desaturation or recycling may occur in certain [^3^H]palmitate pools in response to treatments, which could slightly skew the results obtained only if the resulting palmitate metabolites are selectively or preferentially incorporated by ceramide-producing pathways.

Temporal changes in sphingomyelin levels and ceramide production were also monitored in MCF-7 cells prelabelled to steady-state levels with [^3^H]palmitic acid (1 *μ*Ci ml^−1^) for 24 h. Sphingomyelin was resolved from total cellular lipids by TLC in the solvent system chloroform/methanol/acetic acid/water (50 : 30 : 7 : 4, v v^−1^).

### Expression of data and statistical analysis

Unless otherwise stated, all values are presented as the mean±s.d. from individual representative experiments each carried out in triplicate on two or more occasions. Statistical significance of differences between experimental groups was determined by analysis of variance followed by unpaired Student's *t*-test.

## RESULTS

### Differential action of 4HPR, ATRA and PPARγ agonists on cell growth morphology and survival

**Figure 1:**
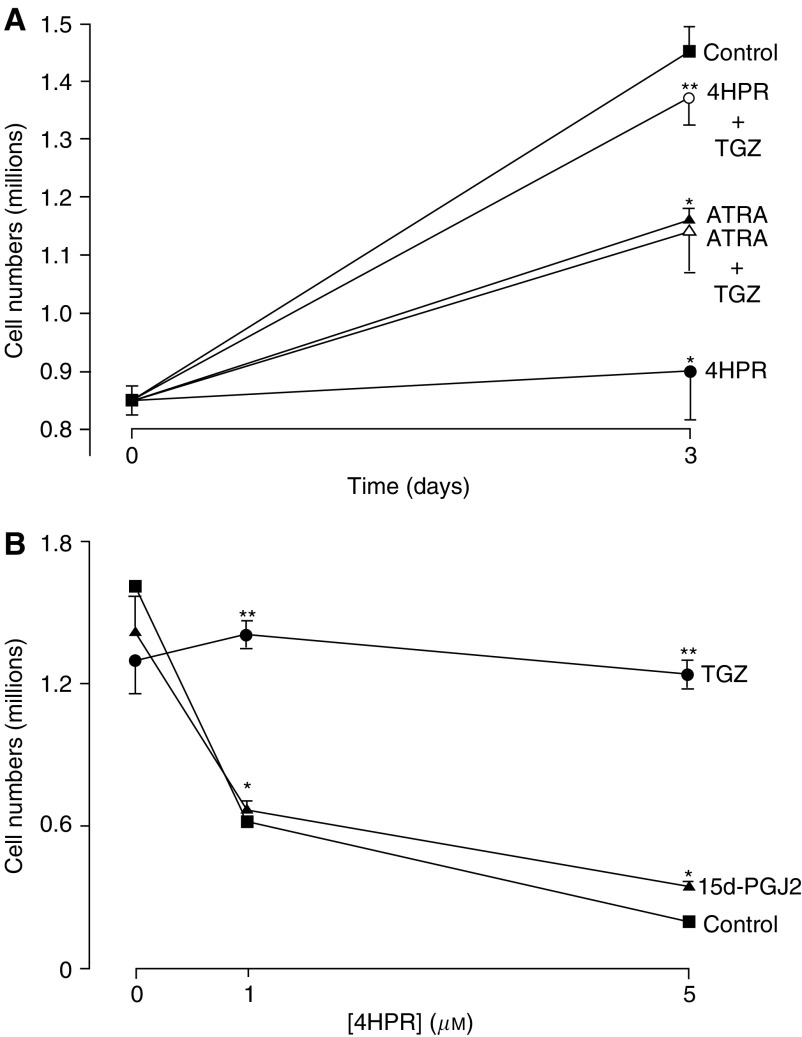
Effect of retinoids and PPAR*γ* agonists on breast cancer cell growth. MCF-7 cells were grown over a 3-day period: (**A**) in the presence or absence of 4HPR, 5 *μ*M; TGZ, 5 *μ*M; or ATRA, 5 *μ*M or (**B**) in the presence or absence of 4HPR; TGZ, 5 *μ*M; or 15d-PGJ_2_), 5 *μ*M. Cell nuclei were harvested and counted as described (see Materials and Methods). (**A**) ^*^P<0.02 for inhibition *vs* control; ^**^P<0.02 for reversal of 4HPR-induced growth inhibition by TGZ. (**B**) ^*^P<0.01 for inhibition *vs* control values in the absence of 4HPR; ^**^P<0.02 for reversal of 4HPR-induced inhibition by TGZ.

**Figure 2:**
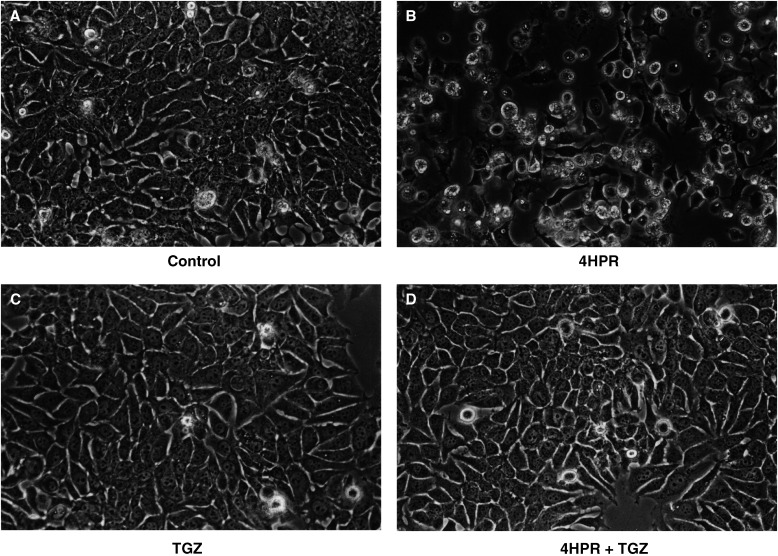
Effect of 4HPR and TGZ on MCF-7 cell morphology. Cells were grown over a 3-day period in the presence or absence of 4HPR, 5 *μ*M and/or TGZ, 5 *μ*M: (**A**) control;(**B**) 4HPR 5 *μ*M; (**C**) TGZ; (**D**) 4HPR and TGZ. Note cell rounding and detachment along with cytoplasmic vacuolation in the presence of 4HPR (**B**). TGZ blocked this 4HPR-induced cytotoxic morphology (**D**).

The RAR agonist ATRA (5 *μ*M) reduced MCF-7 cell growth by around 50% after 72 h, whereas 4HPR (5 *μ*M) prevented any net cell growth during this period (Figure 1A

). The synthetic PPAR*γ* agonist TGZ blocked this action of 4HPR but had no effect on ATRA-induced growth inhibition (Figure 1A). In contrast, the putative PPAR*γ* agonist 15d-PGJ_2_ had no effect on 4HPR action (Figure 1B), suggesting that the interaction of TGZ and 4HPR may be PPAR*γ*-independent. *N*-(4-hydroxphenyl) retinamide also induced a ‘cytotoxic morphology’ with increased cell rounding, vacuolation and detachment, and this action was also blocked by TGZ (Figure 2

). Neither ATRA nor TGZ alone had any significant effect on cell morphology (unpublished observations). Since ROS have been implicated during 4HPR-induced cytotoxicity and TGZ may possess free radical scavenging properties due to its *α*-tocopherol moiety, the effects of the antioxidants *α*-tocopherol and *N*-acetylcysteine on 4HPR action were also investigated. Both *α*-tocopherol (10 *μ*M) and *N*-acetylcysteine (10 mM) partially reversed the inhibitory action of 4HPR on cell growth by 34±3 and 45±5%, respectively (*P*<0.01, *n*=3) in addition to reducing the 4HPR-induced cytotoxic morphology (unpublished observations).

### *N*-(4-hydroxphenyl) retinamide stimulates redox-sensitive ceramide production

**Figure 3:**
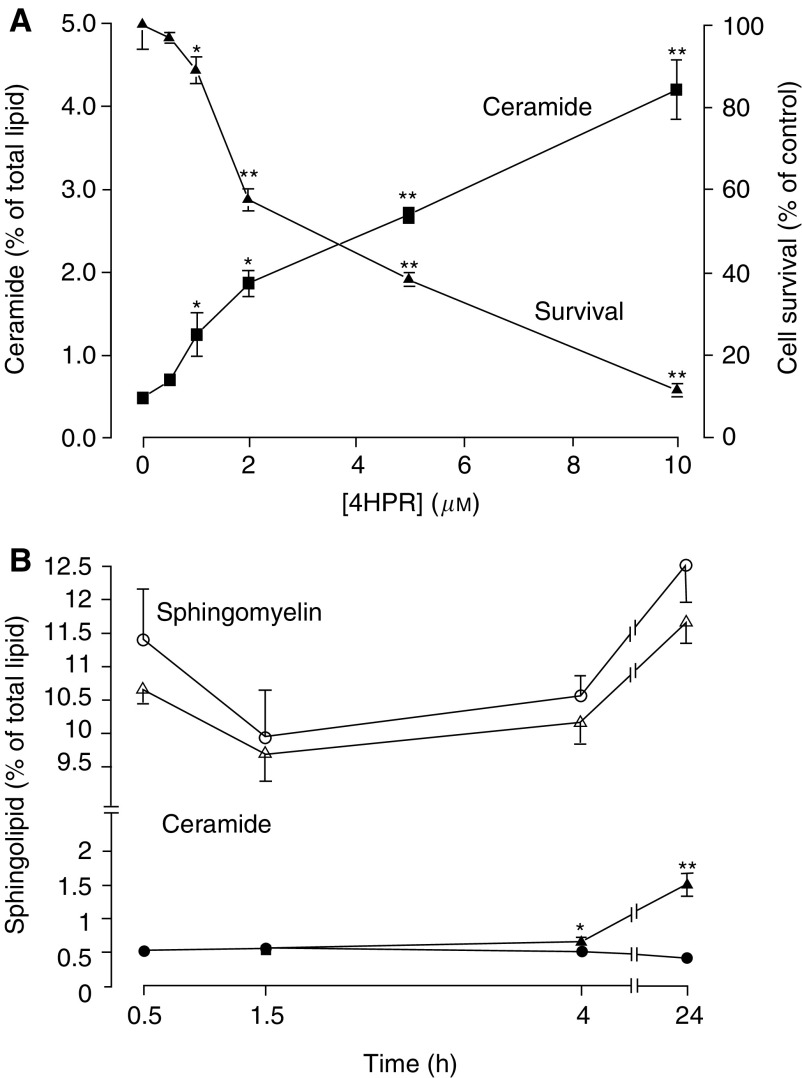
Effect of 4HPR on sphingolipid metabolism and cell survival. (**A**) Cells were grown over a 3-day period in the presence or absence of 4HPR. Cell survival at the end of this time was assessed by the MTS assay as an index of cell viability (see Materials and Methods). Total ceramide production was measured after 24 h as determined by the incorporation of [^3^H]palmitic acid into both ceramide species (C_18_ and C_24_). Radiolabelled ceramide is expressed as a percentage of total ^3^H-labelled cellular lipid, which remained unchanged in response to 4HPR (see Materials and Methods). (**B**) Sphingomyelin levels (open symbols) and ceramide production (closed symbols) were monitored over a 24 h period in the presence (triangles) or absence (circles) of 4HPR (10 *μ*M) in cells previously prelabelled to steady-state levels with [^3^H]palmitate (see Methods and Materials). ^*^P<0.05; ^**^P<0.01 for significant changes in the presence of 4HPR.

**Figure 4:**
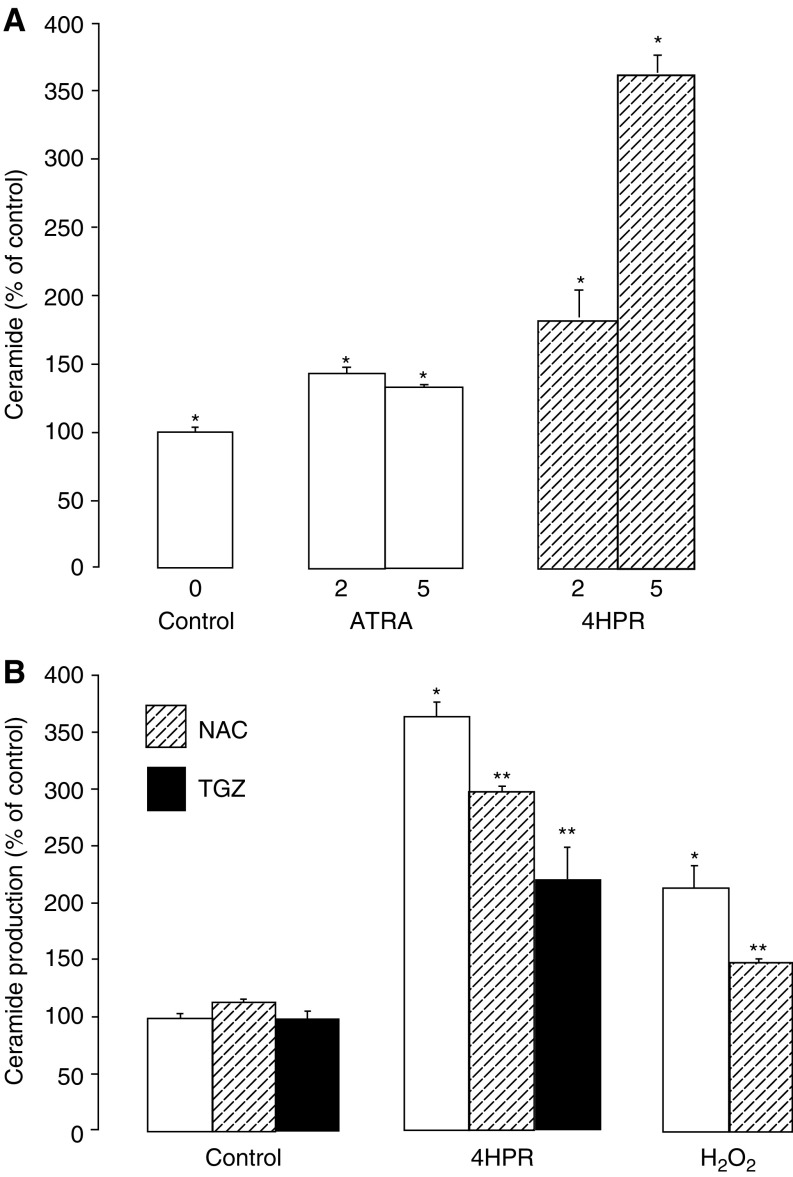
Effect of ATRA, 4HPR and redox modulators on ceramide production. MCF-7 cells were grown to approximately 70% confluence and ceramide production was measured (see Materials and Methods) during a subsequent 24 h period: (**A**) in the absence or presence of ATRA (2, 5 *μ*M) or 4HPR (2, 5 *μ*M); (**B**) in the absence or presence of 5 *μ*M 4HPR, 0.5 mM H_2_O_2_, 10 mM *N*-acetylcysteine (NAC) or 5 *μ*M TGZ. Total ceramide production is expressed as a percentage of control values determined in the absence of 4HPR. Control ceramide levels were calculated to be 0.53±0.04% of total ^3^H-labelled lipid. (**A**) ^*^P<0.02 for stimulation; (**B**) ^*^P<0.02 for stimulation; ^**^P<0.05 for reduction of this stimulation.

The dose-dependent decrease in cell survival after 72 h in response to 4HPR was mirrored by a preceding increase in ceramide production after 24 h (Figure 3A

). Consistent with previous studies, 4HPR also induced an apparent 266% increase in apoptosis as measured by the enrichment of cytosolic nucleosomes after 24 h (see Figure 6). Sphingomyelin hydrolysis has been identified as a mechanism responsible for agonist-induced ceramide production in a number of studies. In MCF-7 cells prelabelled to steady-state levels with [^3^H]palmitate, no significant changes in sphingomyelin levels were detected in response to 4HPR over a 24 h incubation and no major early changes in ceramide production were detected before 24 h (Figure 3B). In contrast to the 3.7-fold increase in cellular ceramide levels observed in response to 4HPR (5 *μ*M), ATRA (5 *μ*M) elicited a relatively modest 40% increase in ceramide (Figure 4A

).

As seen with the effects of 4HPR on cell growth and morphology, TGZ also reduced the ceramide response to 4HPR (Figure 4B) as did the antioxidant *N*-acetylcysteine (Figure 4B). The addition of exogenous oxidant in the form of H_2_O_2_ mimicked the stimulatory effect of 4HPR on ceramide production and similarly, *N*-acetylcysteine also reduced this ceramide response (Figure 4B).

### Effect of putative ceramide modulators on 4HPR action

To further investigate a potential mediatory role for ceramide during 4HPR action, we determined the effects of ceramide synthase activators and inhibitors and glucosylceramide synthase inhibition. The amide-linked fatty acid constituents of mammalian ceramides have acyl chains of varying lengths from 16–24 carbon atoms (C_16_–C_24_). The physiological significance of these different ceramide species in terms of metabolic function or origin is unknown. The putative ‘activator’ of *de novo* ceramide synthesis, PSC 833, stimulated the incorporation of [^3^H]palmitate into both C_18_ and C_24_ ceramide species to similar levels (Table 1

**Table 1:** Effect of 4HPR and PSC 833 on ceramide composition and survival in MCF-7 cells

	**Ceramide composition % of total lipid**		
**Treatment**	**C_18_**	**C_24_**	**Cell survival % of control**
Control	0.21±0.02	0.12±0.02	100±5
4HPR	0.53±0.05*	1.77±0.20*	18±2*
PSC 833	0.82±0.11*	0.81±0.02*	29±3*

4HPR=*N*-(4-hydroxphenyl) retinamide. Ceramide production was measured after 24 h incubation of MCF-7 cells in medium containing [^3^H]palmitic acid in the absence or presence of 4HPR (10 *μ*M) or PSC 833 (10 *μ*M). Incorporation of radiolabelled precursor into the ceramide species C_18_ and C_24_ was determined following lipid extraction and TLC. No significant changes in total cellular ^3^H-labelled lipid were observed in response to any of the treatments, the respective values for control, 4HPR and PSC 833 being 44835±2004, 40816±2092 and 46684±2995 c.p.m. incubation^−1^. Cell survival was measured in separate incubations as described (see Materials and Methods).

* *P*<0.01 for significance *vs* control values.

). In contrast, 4HPR caused a much greater (3.3-fold) accumulation of C_24_ *vs* C_18_ ceramide, an action which was associated with a more potent cytotoxic response to 4HPR (Table 1). These differential actions of 4HPR and PSC 833 are not in keeping with a common mechanism of enhanced ceramide accumulation in response to these agents. Indeed, such differential effects on ceramide molecular species may also reflect a differential action of 4HPR and PSC 833 on palmitate metabolism. The observed effect of PSC 833 in MCF-7 cells is consistent with previous studies (Cabot *et al*, 1998) and lends further support for a mediatory role for endogenous ceramide in the control of cell death.

Recent studies have reported an increased ability of drug-resistant cells to scavenge ceramide via enhanced glycosylation, thereby minimising ceramide-induced apoptotic death (Lavie *et al*, 1996; Nicholson *et al*, 1999). Hence, manipulation of this pathway may modulate chemotoxicity in cancer cells. The effect of the glucosylceramide synthase inhibitor (D,-L,-threo)-1-phenyl-2-decanoylamino-3-morpholino-1-propanol (PDMP) on 4HPR action is shown in Table 2

**Table 2:** Effect of 4HPR and PDMP on sphingolipid metabolism and survival in MCF-7 cells

	**Sphingolipid levels % of total lipid**		
**Treatment**	**Ceramide**	**Glucosylceramide**	**Cell survival % of control**
Control	0.45±0.04	1.42±0.09	100±3
4HPR	1.45±0.43*	1.36±0.07	69±2*
PDMP	0.52±0.04	0.46±0.07*	90±5*
4HPR+PDMP	1.41±0.22*	0.44±0.01*	27±2*

4HPR=*N*-(4-hydroxphenyl) retinamide. PDMP=(D,-L,-threo)-1-phenyl-2-decanoylamino-3-morpholino-1-propanol. Incorporation of [^3^H]palmitic acid into cellular ceramide and glucosylceramide was measured following incubation of MCF-7 cells in the presence or absence of 4HPR (5 *μ*M), PDMP (20 *μ*M) and 4HPR+PDMP as described (see Materials and Methods). No significant changes in total cellular ^3^H-labelled lipid were observed in response to any of the treatments. Cell survival was measured in separate incubations as described (see Materials and Methods).

* *P*<0.02 for significance *vs* control values.

. (D,-L,-threo)-1-phenyl-2-decanoylamino-3-morpholino-1-propanol clearly enhanced the 4HPR-induced decline in cell survival, in addition to reducing glucosylceramide levels by nearly 70% (Table 2). However, the degree of 4HPR-induced ceramide accumulation remained unaltered in the presence of PDMP (Table 2). Furthermore, in contrast to the three-fold increase in ceramide in response to 4HPR, basal glucosylceramide levels remained unaffected by the retinoid (Table 2).

**Figure 5:**
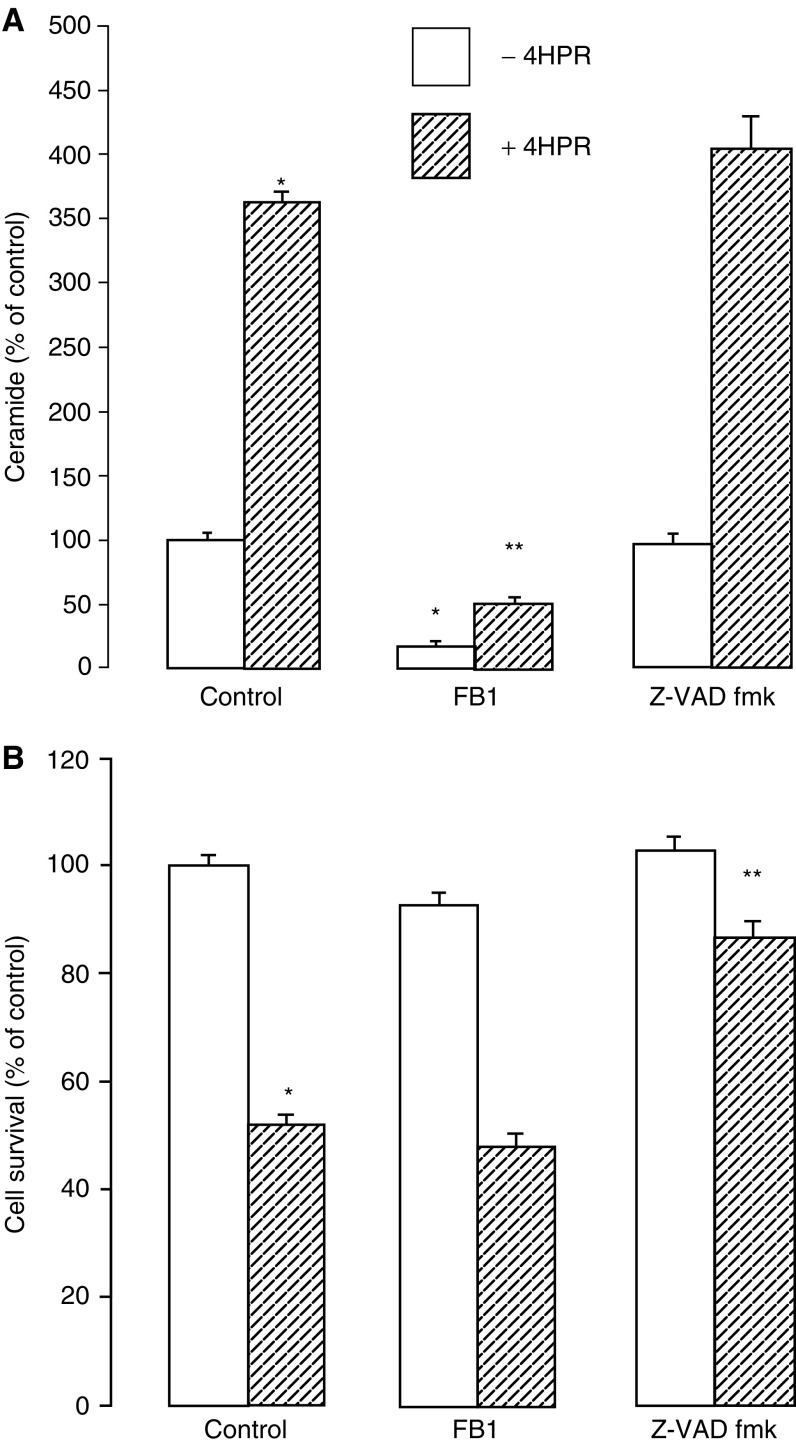
Effects of FB_1_ and caspase inhibition on 4HPR action. MCF-7 cells were grown over a 3-day period in the absence (open bars) or presence (hatched bars) of 4HPR (5 *μ*M), and in the absence or presence of FB_1_ (25 *μ*M) or Z-VAD fmK (50 *μ*M). (**A**) ceramide production was measured after 24 h by incorporation of [^3^H]palmitic acid (see Materials and Methods). (**B**) cell survival was measured after 72 h by the MTS assay (see Materials and Methods). ^*^P<0.01 for significant changes *vs* control values measured in the absence of 4HPR; ^**^P<0.02 for significant changes *vs* control values measured in the presence of 4HPR.

**Figure 6:**
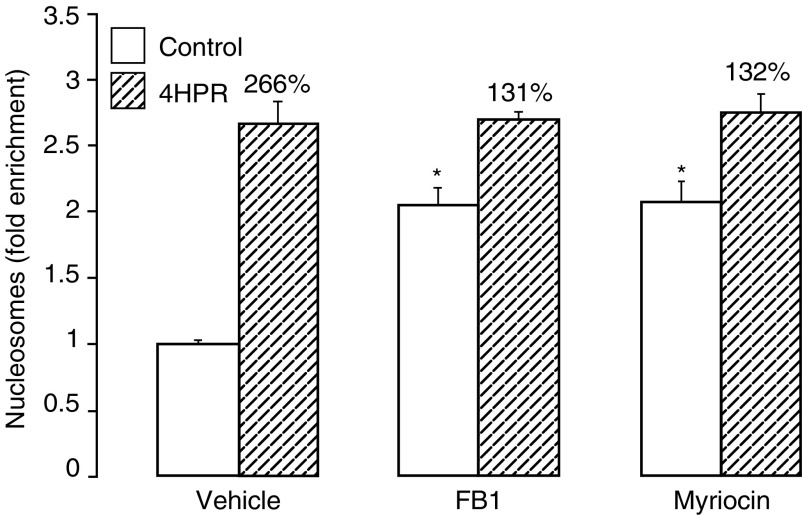
Effects of 4HPR, FB_1_ and myriocin on apoptosis in MCF-7 cells. Cells were pretreated for 1 h with or without FB_1_ (25 *μ*M), myriocin (10 *μ*M) or dimethylsulphoxide vehicle (0.5%), and then incubated for a further 24 h in the presence (hatched bar) or absence (open bars) of 4HPR (5 *μ*M). Apoptosis was determined by ELISA of cytoplasmic DNA–histone complexes and expressed as fold enrichment of nucleosomes compared with untreated controls (see Materials and Methods). Percentage values represent the relative increase in 4HPR-induced apoptosis in the absence or presence of FB_1_ or myriocin. ^*^P<0.02 for significant changes *vs* control values.

The ceramide synthase inhibitor FB_1_ reduced the 4HPR-induced ceramide response by around 90% (Figure 5A

). In contrast, the decline in cell survival after 3 days in the presence of 4HPR was unaffected by FB_1_ (Figure 5B). Similarly, FB_1_ did not prevent the 4HPR-induced cytotoxic morphology (unpublished observations). Although FB_1_ alone did not affect the overall cell survival in control cells after 3 days (Figure 5B), the incorporation of [^3^H]palmitate into ceramide was reduced by around 67% (Figure 5A), and apoptosis was increased two-fold after 24 h (Figure 6

). In relation to respective control values, the apoptotic response to 4HPR (266%) was reduced to 131% in the presence of FB_1_ (Figure 6). Similarly, inhibition of serine palmitoyl transferase, the initial rate-limiting step in ceramide synthesis, by myriocin, which inhibited ceramide synthesis by more than 90% (unpublished observations), also reduced the apparent apoptotic response to 4HPR in addition to inducing apoptosis in the absence of 4HPR (Figure 6). The pancaspase inhibitor Z-VAD fmk partially reversed (by approximately 70%) the decline in cell survival due to 4HPR (Figure 5B), but had no effect on the ceramide response to 4HPR (Figure 5A). In addition to supporting a role for caspases during 4HPR-induced cell death, these observations indicate that ceramide production in response to 4HPR is upstream or independent of caspase activation.

## DISCUSSION

The efficacy of 4-HPR as a chemopreventive agent in premenopausal breast cancer is exemplified by a recent phase III clinical trial, which indicates a reduction both in local recurrence and incidence of contralateral disease in response to treatment (Veronesi *et al*, 1999; Decensi *et al*, 2000). Elucidating the molecular mechanisms involved in this action will be important for the future development and improvement of therapies employing this retinoid in breast cancer treatment.

In agreement with previous studies (Sheikh *et al*, 1995), 4-HPR exhibited a more potent antiproliferative response compared with the native retinoid ATRA, consistent with an additional role for receptor-independent pathways in the action of this synthetic retinoid. In combination with PPAR*γ* agonists, retinoids have been reported to enhance growth inhibition and cell death in human breast cancer cell lines *in vitro* (Elstner *et al*, 1998), possibly via interactive heterodimerisation of retinoid receptors and PPAR*γ*. In contrast, we have observed no such synergism on growth inhibition of MCF-7 cells between ATRA and the synthetic PPAR*γ* agonist TGZ. Indeed, on the contrary, the antiproliferative action of 4-HPR and induction of a cytotoxic morphology were both completely blocked in the presence of TGZ. Furthermore, the putative native PPAR*γ* ligand 15d-PGJ_2_ did not mimic the abrogative action of TGZ, again suggesting a role for receptor-independent interactive mechanisms during the combined actions of 4-HPR and TGZ. This is further supported by the antagonism of 4-HPR-induced growth inhibition and cytotoxicity by the antioxidants *α*-tocopherol and *N*-acetylcysteine. Given that TGZ also possesses antioxidant properties by virtue of its constituent *α*-tocopherol moiety, a regulatory function for cellular redox status can be invoked during 4-HPR action in MCF-7 cells in the present study.

*N*-(4-hydroxphenyl) retinamide induced a dose-dependent decline in cell survival, which was associated with a corresponding reciprocal dose-related increase in ceramide accumulation. Ceramide production in response to 4-HPR was an order of magnitude greater than that observed in the presence of ATRA, again consistent with a possible receptor-independent regulation of ceramide in response to 4-HPR. One potential mediatory mechanism for this action could be via the modulation of cellular redox status and/or generation of ROS, since the antioxidant *N*-acetylcysteine and the radical scavenger TGZ both reduced this ceramide response to 4-HPR. In addition, the exogenous oxidant H_2_O_2_ mimicked the stimulatory action of 4-HPR on ceramide production. Although neutral sphingomyelinase has been previously proposed as a downstream target of oxidative stress (Andrieu-Abadie *et al*, 2001), we found no evidence of a role for sphingomyelin hydrolysis as a metabolic source for ceramide during 4-HPR action. Indeed, inhibition of this response to 4-HPR by FB_1_ and myriocin indicates a stimulatory action on the *de novo* synthesis pathways of ceramide production as the mechanism mediating this increase in ceramide levels.

Despite this effective blockade of the ceramide response to 4-HPR by FB_1_, the 4-HPR-induced decline in cell survival after 72 h was not prevented. Although there is thus an apparent dissociation of a mediatory role for ceramide during this action of 4-HPR, a dilemma often pertains when employing pharmacological manipulation of sphingolipid metabolism to investigate a functional role for ceramide. As essential structural and functional membrane components in growing cells, there is a continual dynamic turnover of sphingolipids as well as an ongoing requirement for their *de novo* synthesis. Compromising these pathways with enzyme inhibitors such as FB_1_ and myriocin may also lead to adverse cytotoxicity and apoptosis (Nakamura *et al*, 1996; Schmelz *et al*, 1998). Indeed, both agents induced apoptosis in MCF-7 cells in the present study while paradoxically appearing to reduce the apoptotic response to 4-HPR. Furthermore, inhibition of ceramide synthase by FB_1_ may lead to the accumulation of sphinganine, and other sphingoid bases, which in turn could cause inhibition of cell growth and induction of apoptosis (Schmelz *et al*, 1998).

The metabolic fate of endogenous ceramide production may also be subjected to pharmacological modulation. Enhanced glycosylation of ceramide by glucosylceramide synthase may contribute to multidrug resistance in cancer cells by abrogation of ceramide-mediated cell death signalling (Lavie *et al*, 1996). In this respect, transformed cells exhibit a synergistic increase in cytotoxicity in response to 4-HPR in the presence of glucosylceramide synthase inhibitors (Nicholson *et al*, 1999). In the present study such an inhibitor, PDMP, did indeed enhance the decline in cell survival in response to 4-HPR. However, such findings must be interpreted with caution, since while inhibiting basal glycosylceramide production, PDMP did not modify the 4-HPR-induced ceramide response. Furthermore, it has been recently reported that PDMP also inhibits nucleoside transport independent of ceramide production (Griner and Bollag, 2000). It thus remains equivocal as to whether glucosylceramide synthase plays a major role in modulating a putative drug-sensitive ceramide pool during 4-HPR action in MCF-7 cells. Indeed, the 4-HPR-sensitive ceramide pool appeared not to be in short-term metabolic equilibrium with that of glucosylceramide, since levels of the latter sphingolipid remained unchanged in response to 4-HPR. In contrast, under the same conditions, stimulation of *de novo* ceramide production in MCF-7 cells by PSC 833 led to increases in both ceramide and glycosylceramide (Cabot *et al*, 1998).

We have employed PSC 833, which stimulates the rate-limiting step, serine palmitoyltransferase (Wang *et al*, 2002), ostensibly as a positive control in the activation of *de novo* ceramide production. A differential mechanism of action for the stimulation of *de novo* ceramide synthesis by PSC 833 and 4-HPR in the present study was also evident in the qualitative and quantitative nature of the ceramide species produced; the major species produced being C_18_ or C_24_ in response to PSC 833 or 4-HPR, respectively. The functional significance of these differences in relation to an active role for ceramide during drug-induced cytotoxicity is unknown. However, it is interesting to note that an increased accumulation of C_24_ ceramide was associated with a more potent loss of cell survival in response to 4-HPR *vs* PSC 833.

Mechanistic differences in cell signalling in response to 4-HPR and PSC 833 also include opposing actions on cellular redox. Several studies have reported the elevation of ROS in response to 4-HPR in various cell types (Sun *et al*, 1999) and in this context, we have already proposed a mediatory role for oxidative stress during 4-HPR action on ceramide production and cell survival in the present study; on the other hand, PSC 833 has been reported to suppress the generation of ROS (Nguyen *et al*, 1998). A role for ceramide has been recently implicated during nitric oxide-induced cell death (Rabkin, 2002) and 4-HPR is known to induce nitric oxide synthase in breast cancer cells (Simeone *et al*, 2002). However, in a preliminary study employing different nitric oxide synthase inhibitors, we found no evidence of a mediatory role for nitric oxide during 4-HPR-induced ceramide production in breast cancer cells (Abbara and Schrey, 2003).

The relationship of caspase activation and ceramide production with respect to 4-HPR action is unclear. Caspase-3 may partially mediate 4-HPR-induced apoptosis in some cell types (Perry, 2000) and ceramide can activate caspase-3 (Mizushima *et al*, 1996); a potential mediatory role for ceramide can thus be hypothesised during drug-induced caspase activation. However, since MCF-7 cells express a mutant nonfunctional caspase-3 due to a 47 base pair deletion in exon 3 of the CASP-3 gene (Janicke *et al*, 1998), such a role is unlikely in the present instance. Furthermore, in the case of caspase-3-dependent apoptosis induction by daunorubicin in lymphoblastic leukaemia cells, ceramide synthase appears to be a downstream target of caspase action (Turnbull *et al*, 1999). In the present study, the effect of the pancaspase inhibitor Z-VAD fmk suggests that at least 70% of the decline in MCF-7 cell survival in response to 4-HPR is caspase-dependent. On the other hand, ceramide production in response to 4-HPR was unaffected by Z-VAD fmk, indicating it to be either upstream or independent of caspase activation.

Consistent with findings described above on 4-HPR action in MCF-7 cells, in the case of MDA MB 231 breast cancer cells we have recently observed a similar paradoxical differential action of both FB_1_ and myriocin on overall cell survival *vs* apoptosis. Thus, the decline in cell survival in response to 4-HPR after 72 h is unaffected by blockade of ceramide production, whereas apoptosis monitored after 24 h is partially prevented (Abbara and Schrey, 2003). Such observations illustrate the complex manifold actions of 4-HPR, which may cause cell death by mixed apoptosis/necrosis by both caspase-dependent and -independent pathways (DiPietrantonio *et al*, 1998; Maurer *et al*, 1999). Indeed, in contrast to the caspase-dependent nature of 4-HPR action on MCF-7 survival described in the present study, caspase activation was not required for 4-HPR-induced cytotoxicity in MDA MB 231 cells (unpublished observations).

The present study describes a novel redox-sensitive stimulation of ceramide production in breast cancer cells by 4-HPR. Although elevation of this sphingolipid is associated with the cytotoxic action of 4-HPR, a major mediatory role for ceramide in this context remains equivocal. Our findings also highlight a need for caution when interpreting a functional role for ceramide signalling during 4-HPR action, when it is based on the effects of putative modulators of ceramide metabolism.
